# Synchronous Lymphoma: Diagnostic Challenges in a Case of Coexisting Diffuse Large B-cell Lymphoma and Classical Hodgkin Lymphoma

**DOI:** 10.7759/cureus.78248

**Published:** 2025-01-30

**Authors:** Farah Rana, Minakshi Mishra, Amitabh Kumar Upadhyay, Abhishek Kumar, Radhika Narayan

**Affiliations:** 1 Pathology, Tata Main Hospital, Jamshedpur, IND; 2 Medical Oncology, Tata Main Hospital, Jamshedpur, IND; 3 Nuclear Medicine, Tata Main Hospital, Jamshedpur, IND

**Keywords:** classical hodgkin lymphoma, diffuse large b cell lymphoma, diffuse large b-cell lymphoma, gastric biopsy, immunohistochemistry, inguinal lymph node, mixed cellularity chl, synchronous lymphoma

## Abstract

Synchronous lymphoma (SL) is the simultaneous occurrence of two or more unique kinds of lymphomas in the same individual at the initial diagnosis. Accurate diagnosis requires histopathological examination, immunohistochemistry (IHC), and molecular studies. We found only a handful of reported cases of SLs involving different anatomical sites in medical literature, and no large-scale studies have specifically addressed the frequency of this combination. Given the rarity of this combination, each case is often reported as a unique case study, and treatment approaches may vary depending on individual circumstances. Here, we report a case of a 60-year-old female patient presenting with pain in the abdomen, vomiting, fever, significant weight loss, and palpable inguinal nodes. Her endoscopic biopsy from the stomach revealed diffuse large B-cell lymphoma (DLBCL) while the inguinal node biopsy showed classical Hodgkin lymphoma (cHL), mixed cellularity type. Our report discusses and highlights the importance of detailed clinical history, investigations, and molecular workups that are essential to diagnosing these rare cases.

## Introduction

Diffuse large B-cell lymphoma (DLBCL) is a mature B-cell neoplasm. While classical Hodgkin lymphoma (cHL) is also a neoplasm derived from B-cells, they have different immunophenotypes and genetic characteristics. Although it is rare for these two distinct forms of lymphoma to be found together in the same lesion (composite lymphomas (CLs)), it may occur more frequently than what is currently reported in the literature. The transformation of different kinds of indolent lymphomas, including splenic marginal zone lymphoma, marginal zone (mucosa-assisted lymphoid tissue (MALT)) lymphoma, and chronic lymphocytic leukemia (Richter transformation), can result in DLBCL and rarely (<0.5%) into CLs or sequential lymphomas (SLs) of other histology [1,2]. Concurrent and sequential DLBCL and indolent lymphomas are reported to have an incidence of 12.9% [3]. Reported cases in published literature demonstrate that the co-occurrence of cHL and non-Hodgkin lymphoma (NHL) is a less extensively reported occurrence [4-7].

Composite synchronous lymphomas that appear within a single lymph node are comparatively more straightforward to identify by lymph node removal and histological evaluation. On the other hand, detecting synchronous or discordant lymphomas that arise in different anatomical areas is only possible by collecting samples from several sites. This approach is not very prevalent in standard clinical settings, especially when the patient's clinical presentation is consistent with a verified diagnosis of a lymphoproliferative illness. This could be the reason that only a tiny percentage of cases with synchronous lymphomas at different sites are identified. Nodular lymphocyte-predominant Hodgkin lymphomas (NLPHL), B-cell lymphomas (including follicular, mantle, and marginal zone lymphomas along with DLBCL), and T-cell lymphomas have all been linked to cHL. However, synchronous DLBCL and cHL are extremely rare occurrences. At first, it was thought that two distinct morphological diseases were coincidentally associated with discordant lymphomas, but both contingents of these lymphomas have been shown to share molecular abnormalities [4]. Recent investigations have identified several common factors, including (i) common gene translocations such as CCND1, BCL6, and BCL2; (ii) common rearrangements of the immunoglobulin heavy (IgH) and light (Igκ) chains; and (iii) frequent pathogenic variants, including BCOR, KMT2D, SF3B1, TP53, BCL2, and ARID1A, among different groups [8].

It has been proposed that transdifferentiation phenomena in CLs could be caused by the plasticity of mature B-cells [9]. Studies have revealed that secondary NHL may occur both synchronously and sequentially with cHL. Synchronous NHL is reported to have a worse prognosis compared to sequential cases when the different lymphomas have not been recognized at the time of diagnosis [3]. Our case highlights difficulties when encountering such a case in diagnostic laboratories with limited resources.

## Case presentation

A female patient in her 60s, without any comorbidities, presented with a history of chronic cough, pain in the abdomen, vomiting, and loss of appetite at our hospital. The patient experienced significant weight loss and intermittent fever over the past three months. She did not give any history of Koch’s infection, chronic cough, gastrointestinal disorders, or cancers in her family. During a general examination, she was found to have severe pallor and a palpable right inguinal lymph node measuring approximately 3 cm.

She gave a history of a proliferative ulcer on the right side of the hard palate, which was biopsied and later excised on suspicion of neoplastic pathology six months prior to the current presentation. A review of the oral biopsy showed a predominantly monomorphic lymphoid population with few interspersed histiocytes and a fair number of congested blood vessels without any defined capsule. Lymphoid follicles or germinal centers were not appreciated. There was no evidence of epithelial dysplasia or malignancy. It was suggested to be of reactive/inflammatory pathology with the advice of an immunohistochemistry test on the biopsy to exclude a mucosal low-grade clonal lymphoproliferative disorder. However, the patient was lost to follow-up, and the immunohistochemistry (IHC) workup was not done on this biopsy.

Her complete blood counts revealed hemoglobin (Hb) of 7.5 gm/dl, a total leukocyte count of 14,180 cells/cu mm, with 87% neutrophils, and a platelet count of 574,000/cu mm. Her renal and liver function tests were reported to be within normal ranges. Her chest X-ray showed bilateral nodular lesions suggestive of metastasis. The biochemical investigations revealed the following: CA19-9: 14.4 U/ml; CA-125: 327.2 U/ml, AFP: 1.78 ng/ml, CEA: 1.27 ng/ml. Routine viral serological markers for human immunodeficiency virus (HIV) I and II, hepatitis C virus (HCV), and hepatitis B surface antigen (HBsAg) were negative.

**Figure 1:**
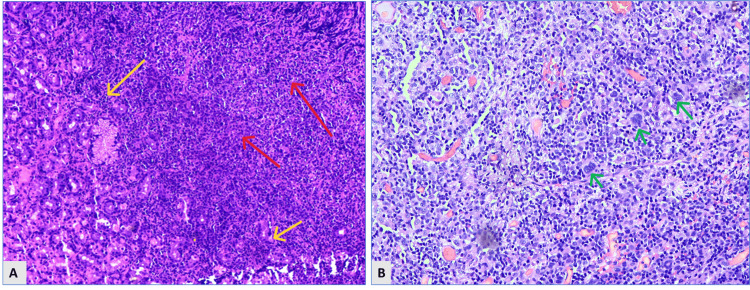
A: Hematoxylin and eosin-stained photomicrograph of gastric mucosal biopsy section showing diffuse sheets of lymphoma cells infiltrating between the gastric glands and lamina propria (yellow arrows), causing wide separation and disrupting the native architecture (red arrows). They have round, oval, or irregular nuclei, multiple punctate nucleoli, and scant cytoplasm. B: Hematoxylin and eosin-stained photomicrograph of an inguinal lymph node section showing scattered large Reed-Sternberg (RS) cells and Hodgkin cells (green arrows) in a reactive microenvironment composed of lymphocytes, eosinophils, neutrophils, plasma cells, histiocytes, and fibroblasts (hematoxylin and eosin, 40X).

The upper GI endoscopy revealed diffuse gastric ulcers with thickened walls, which prompted a gastric mucosal biopsy from the ulcerated area. It showed gastric mucosal tissue fragments infiltrated by monomorphic medium to large lymphoid cells in sheets having nuclear membrane irregularity and conspicuous nucleoli. Brisk mitosis, apoptosis, and focal crushing suggested a high-grade NHL (Figure 1A).

A day after the endoscopic procedure, an excisional biopsy of the right inguinal lymph node was also performed. It grossly measured 3.5 x 3 x 2 cm, was firm in consistency, and had a homogenous cream-white cut surface. On microscopic examination, sections from the node showed a polymorphous infiltrate of cells comprising mature lymphocytes, eosinophils, neutrophils, histiocytes, and plasma cells. Occasional scattered large atypical mononuclear cells with variably prominent eosinophilic nucleoli were identified, raising the possibility of cHL (Figure 1B).

**Figure 2:**
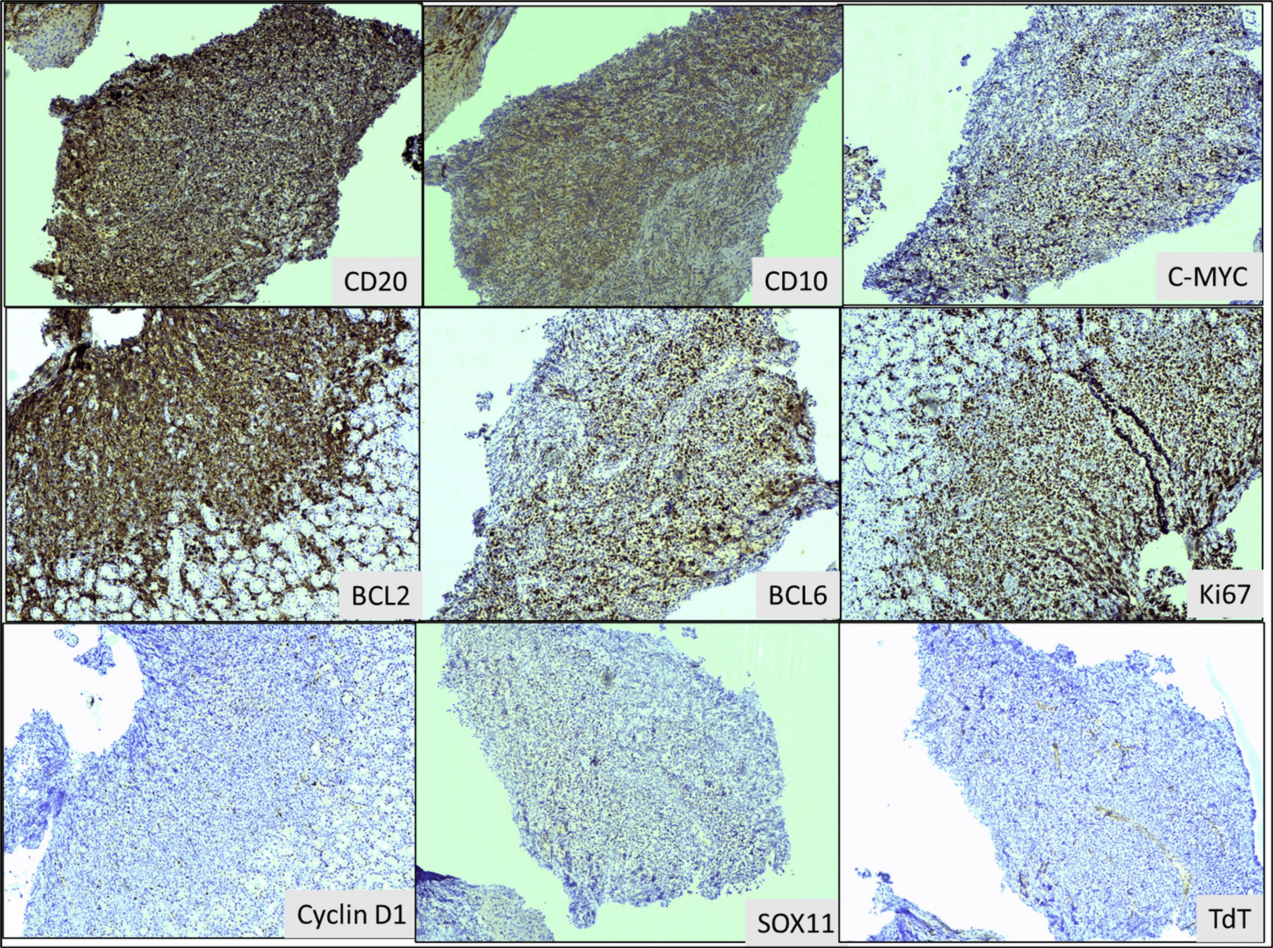
Immunohistochemistry images of gastric biopsy showing diffuse, strong positive staining for CD20 (100X) along with positive staining for CD10 (100X), C-MYC (100X), BCL2 (100X), and BCL6 (100X), along with a >90% Ki67 proliferative index (100X), favoring high-grade B-cell lymphoma, double expressor phenotype. Negative staining for cyclin D1 (100X), SOX11 (100X), and TdT (100X) ruled out other large cell lymphomas.

Immunohistochemistry on the gastric biopsy showed that the lymphoid cells were diffusely positive for CD20. These cells were also positive for CD10, BCL2, BCL6, and c-MYC (60% of cells were positive). The cells were negative for CD3, CD23, cyclin D1, Tdt, SOX11, and MUM1. The proliferation index for Ki-67 was almost 90%. Background T-cells had been highlighted with CD3 (Figure 2). A diagnosis of high-grade B-cell lymphoma, favoring DLBCL, germinal-center B-cell type (GCB), and double expressor immunophenotype was made, and fluorescence in situ hybridization (FISH) was suggested to rule out or confirm a double/triple-hit lymphoma.

**Figure 3:**
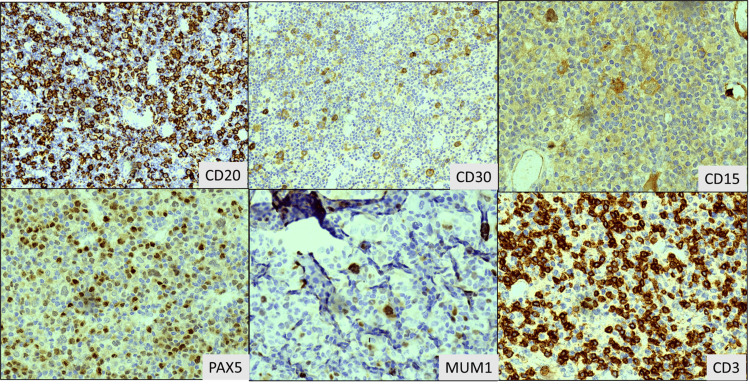
Immunohistochemistry images of inguinal lymph node biopsy showing heterogeneous staining for CD20 (200X). CD30 positivity (100X), patchy CD15 positivity (400X), along with weak PAX5 (400X) and positive MUM1 (400X) in larger cells. CD3 was positive in background T cells (400X), favoring Hodgkin lymphoma, classical type with Reed-Sternberg (RS) cells.

The inguinal lymph node's IHC revealed that the larger cells were heterogeneously stained with CD20, patchy positivity for CD15 was found, and they were positive for MUM1, PAX5, and CD30. The cells were negative for BCL6 and CD3. CD3 highlighted the background reactive T-cells. Hence, it was diagnosed as cHL (Figure 3).

**Figure 4:**
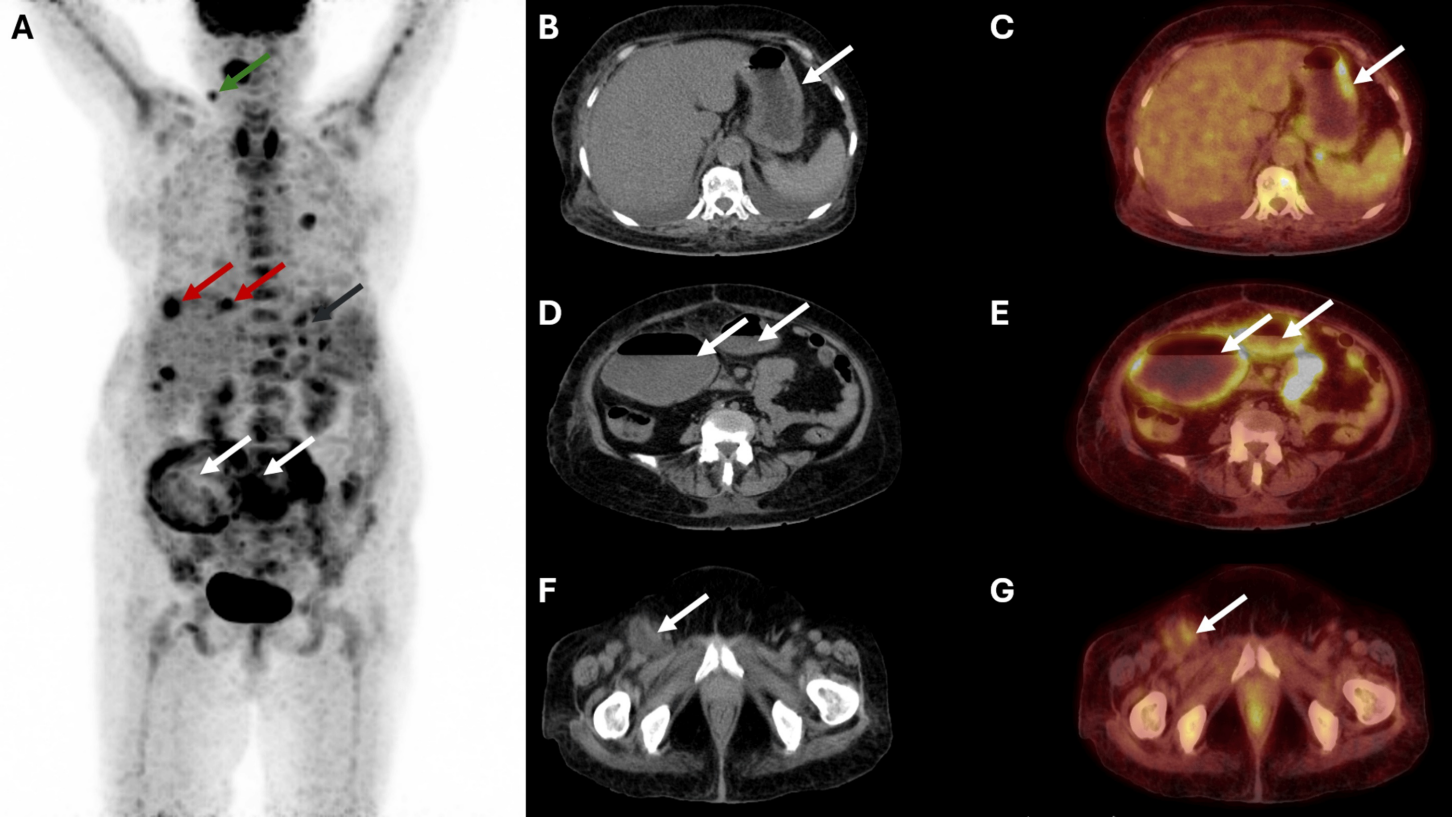
PET-CT images Maximum intensity projection (MIP) image (A) reveals a nodal deposit in the neck (green arrow). Deposits in the region of the liver (red arrows) and stomach (black arrow) are also noted. FDG uptake is noted in the region of the bowel loops (white arrows) in the abdomen. The CT image (B) and fused PET-CT image (C) reveal FDG-avid gastric wall thickening (white arrow). CT image (D) and fused PET-CT image (E) reveal FDG avid wall thickening involving dilated small bowel loops (white arrows). CT image (F) and fused PET-CT image (G) reveal a lymph node in the right inguinal region with evidence of necrosis (white arrow). FGD: fludeoxyglucose-18; PET-CT: positron emission tomography-computed tomography

Whole-body fludeoxyglucose-18 (FDG) positron emission tomography-computed tomography (PET-CT) scan done after the morphological diagnosis showed hypermetabolic wall thickening involving the stomach and FDG-avid circumferential wall thickening involving jejunal bowel loops with aneurysmal dilatation and adjacent fat standing. Overall, these findings also suggested a lymphomatous involvement. Multiple hypermetabolic lymph nodes were seen in the submandibular, bilateral internal mammary, bilateral anterior diaphragmatic, retroperitoneal regions, and right inguinal region. There was evidence of minimal ascites and moderate bilateral pleural effusion and consolidation in the bilateral lung. There was a defined deposit in the region of the surface of segment VIII of the liver. Diffuse uptake in narrow spaces of the visualized skeleton and spleen suggested diffuse lymphomatous involvement (Figure 4).

Her International Prognostic Index (IPI) had five points, and it classified her into poor prognosis (Revised International Prognostic Index (R-IPI)) and high-risk group (IPI).

At this stage, a diagnosis of synchronous DLBCL with nodal cHL was made. The possibility of a cHL transforming to a DLBCL or an indolent lymphoma with progression and discordant differentiation was also considered.

Subsequently, Epstein-Barr virus early RNA in situ hybridization (EBER ISH), FISH, and IgH chain fragment length analysis and next-generation sequencing (NGS) were suggested to investigate the case further and establish the cell of origin to arrive at a possible explanation for the discordant lymphomas. However, tissue availability (after the extensive IHC panel at an outside reference lab facility), financial constraints, and patients' clinical conditions halted further planned tests.

The patient was experiencing symptoms of acute severe abdominal pain, recurrent vomiting, and signs of impending small intestinal obstruction. Based on the histomorphology report from the laboratory (while her IHC reports were still pending from the referral center), taking into account her high disease burden (as shown by PET-CT findings), deteriorating general condition, and severe symptoms, she was advised to undergo pre-phase chemotherapy with cyclophosphamide, vincristine, and steroids. This treatment was planned to be carried out under broad-spectrum antibiotics. Her body surface area was 1.45 m², and she received a lower flat dose of cyclophosphamide 800 mg, vincristine 1.5 mg, and dexamethasone 40 mg, considering her general condition. It was planned to escalate to the standard regime and to add rituximab after IHC confirmation. The patient experienced vomiting, fever, and weakness after receiving chemotherapy. She was treated with a combination of broad-spectrum antibiotics and antifungal therapy. Although she could consume semisolid foods and pass stools normally, her complete blood count gradually decreased with the total leukocyte count (TLC) reaching a nadir of 130 cells/cu mm on Day 7. Treatment continued with broad-spectrum antibiotics, daily granulocyte colony-stimulating factors injections (GCSF), and supportive measures.

Gram staining of her sputum revealed the presence of Gram-negative bacilli, but acid-fast bacilli were not detected. Her blood and urine cultures were sterile, while her sputum culture showed the presence of coagulase-negative, catalase, and oxidase-positive Klebsiella, which was found to be sensitive to meropenem, colistin, and nitrofurantoin. She gradually improved with sensitive antibiotics, GCSF, and other supportive measures. Her TLC improved to 1360 cells/cu mm on day 10,9620 cells/cu mm on Day 11, and 11,900 cells/cu mm on Day 12 post chemotherapy. There had been no thrombocytopenia with a nadir platelet count of 150,000/cu mm.

However, she suddenly collapsed with unrecordable blood pressure (BP) and pulse on the evening of Day 12 and could not be resuscitated despite best efforts. A possible cardiac event was assumed but could not be proven in her case.

## Discussion

Most human lymphomas exhibit uniform differentiation. But unlike Hodgkin lymphomas, NHL is a varied collection of lymphoproliferative cancers that are considerably less predictable. Non-Hodgkin lymphoma has a considerably greater propensity to migrate to extranodal tissues and histologically advance to a higher grade [10]. The pathogenic type is essentially the same and remains constant throughout the development and progression of the disease.

On the other hand, of the two primary subtypes of Hodgkin lymphoma, cHL and NLPHL, NLPHL has a slightly greater chance of transformation into aggressive DLBCL, a type of NHL [11]. Although less frequent, a substantial correlation exists between NHL and nodular sclerosis or mixed cellularity Hodgkin's disease. According to investigations, most NHL linked to Hodgkin's disease are B-cell-derived, most frequently follicular lymphomas. They are usually Epstein-Barr virus (EBV)-positive when associated with CLs and EBV-negative when arising after treatment of HD. The conversion of cHL to NHL or vice versa, on the other hand, is an infrequent incidence that happens in only 1% to 2% of instances [4].

Diffuse large B-cell lymphoma is histologically comprised of intermediate to large B cells in a diffuse growth pattern effacing the normal architecture and may be accompanied by fibrosis or necrosis. Prominent single-cell apoptosis with a high mitotic rate is usually seen with a background of reactive T lymphocytes and histiocytes. However, DLBCL can have variant morphology like centroblastic, immunoblastic, and anaplastic variants. Of these, the less commonly seen anaplastic variant shows large neoplastic cells with pleomorphic nuclei, mimicking Reed-Sternberg (RS) cells of Hodgkin lymphoma [12].

Our patient was a middle-aged female with GI involvement due to diffuse large ulcers and B-symptoms. The biopsy showed a characteristic diffuse infiltrative growth pattern by intermediate- to large-sized lymphocytes with prominent nucleoli and showed an antigenic expression pattern of B-cell-derived lymphoma with diffuse strong positivity for CD20. CD10, BCL2, and BCL6.C-MYC were also positive. As the neoplastic cells were negative for CD3, CD23, cyclin D1, Tdt, SOX11, and MUM1, the mantle and marginal zone lymphomas that can occur at this site were ruled out. A high proliferative index represented by Ki67 positivity favored a high-grade B-cell lymphoma with MYC, BCL2, and BCL6 rearrangement, possibly a double-expressor phenotype or a triple hit lymphoma if proven by classic cytogenetics, FISH, or other molecular or genetic tests.

The nodal involvement in our case needed to be distinguished from DLBCL with variant morphology. The histopathology of the node showed Hodgkin and Reed-Sternberg (HRS) cells in a mixed inflammatory background. To differentiate HRS from RS-like cells, IHC was important. B-cell markers are known to be rarely expressed in cHL with MUM1 being a sensitive marker for HRS cells in cHL. The HRS cells are PAX5+ (dim), CD30+, CD15 variable, and CD20 variable, as was seen in our case. It was important to identify the IHC pattern as distinct from that of high-grade B-cell lymphoma to avoid these diagnostic pitfalls and ultimately to reach an accurate diagnosis [13].

In situ hybridization for EBV (EBER) positivity in larger cells and immunoglobulin gene rearrangement studies by polymerase chain reaction (PCR) would have further helped in confirming Hodgkin lymphoma type. The restriction of the cytogenetic abnormality to HRS cells would have further strengthened the diagnosis of CHL in the inguinal node of this patient [14]. Extranodal involvement is commonly seen in high-grade DLBCL, while B symptoms before the appearance of nodal enlargements favor cHL [15]. The rarity of the case reports with common clinical features of nodal or extranodal lymphomatous involvement, coupled with similar B-symptoms of unexplained fever, cough, weight loss, and night sweats, prevents a suspicion of dual pathology at the outset. 

Resource constraints with IHC being outsourced initially, compounded by the sudden death of our patient, prevented further molecular testing in our setting. The lack of advanced molecular studies in our case reports, such as FISH, PCR, next-generation sequencing, or IgH chain clonality analysis, limits the determination of any potential clonal relationships between the two lymphomas. Taking everything into account, it is impossible to rule out clone-related lesions in this situation.

Although cHL is a treatable condition, survivors are at an elevated danger of evolving second malignancy. After cHL is first treated, NHL is more often found to be a secondary neoplasm when a biopsy is performed because of a potential relapse or refractoriness of the disease. Treatment-related immunodeficiency, immunoregulatory abnormalities brought on by cHL itself, or the negative consequences of chemotherapy and radiation therapy can all be blamed for this substantial danger of SL development when cHL develops first, then large B cell lymphoma (LBCL) (cHL-LBCL), or vice versa (LBCL-cHL) [16]. In large cohort studies, NHL cumulative prevalence rates after cHL ranged from 0.9% to 1.5% over 10 years. There was no discernible variation in survival among patients who developed aggressive NHL following cHL with their de novo aggressive NHL counterparts, following an investigation that included instances with cHL diagnosed between 1992 and 2009 from the Surveillance, Epidemiology, and End Results (SEER) database [17-21]. Both synchronous and sequential secondary NHL symptoms may appear after cHL. Studies have also shown that NHL manifestation can occur at various times: as part of primary refractory cHL or following effective cHL treatment, with at least 18 months of total remission. Notably, clonal connections among cHL and NHL in the primary refractory instances have not been found in some studies. However, in a few cases, NGS data has suggested that NHL might be synchronous. Among patients with successively occurring NHL, the clonal association between cHL and secondary DLBCL has been confirmed by the IgHV gene’s PCR testing, suggesting possible clonality among the two lymphomas [3,22,23]. The study findings also demonstrate that when discordant lymphomas are not detected at diagnosis, synchronous NHL's prognosis is worse than sequential NHL's. More EBV identifications have been made in Hodgkin's disease and composite NHL. Sometimes, it is possible to identify mutations accompanying neoplastic cells during their clonal growth, with more mutations appearing later. The spatial heterogeneity of lymphoid malignancies can be resolved by analyzing liquid biopsy specimens utilizing NGS. This identification may eventually result in early patient selection & alternate treatment approaches, which might boost the probability of recovery [3,4].

Composite lymphoma shares similarities with malignant gray zone lymphoma with regard to clinical presentation and prognosis. The treatment consequences and available treatments for CL with cHL and DLBCL/primary mediastinal B-cell lymphoma (PMBCL)(LBCL) are yet unknown because few studies have been conducted on these conditions [24-26]. It is also worth noting that the diagnosis of synchronous or sequential discordant lymphomas can be difficult based only on morphology, as our case demonstrated. Different subtypes or clones may have different morphological, immunophenotypic, and genetic features. Accurate diagnosis can only be made after careful histopathological examination, immunophenotyping or immunohistochemical confirmation, and molecular studies to establish a single or different clonality.

A literature search revealed only an occasional case report of SLs occurring at different anatomical sites. Most cases reported in the literature are of composite synchronous lymphomas occurring at the same anatomical location [27].

Kim et al. in 1977 described CLs in a series of 20 cases. In this series, in 12 patients, the CLs consisted of different types of NHLs, whereas a combination of Hodgkin's disease and NHL was observed in the remainder. The composite pattern was present in lymph nodes in 12 patients, the spleen in three, the retroperitoneum in one, the liver in two, and both the spleen and lymph nodes in two patients. They also highlighted that prognosis related to the recognizably more aggressive component [28].

In 2016, Gaurav et al. reported a case of a composite lymphoma with DLBCL, not otherwise specified (NOS), and cHL involving the terminal ileum, colon, and pericolic lymph nodes. Immunohistochemistry showed a germinal center B-cell subtype, while IgH chain fragment length analysis indicated a partial clonal relationship between the two components. They suggested that the two components of CL with DLBCL-NOS and cHL components are clonally related and suggest a shared origin from a common B-cell precursor [29].

In 2017, Zhang and colleagues reported an instance of discordant lymphomas in a 50-year-old man who had mixed cellularity Hodgkin lymphoma in the pyloric group lymph nodes and gastric pylorus at the same time as simultaneous gastric antral DLBCL. In their reported case, the morphologies and immunophenotypes of the two lymphomas were inconsistent, and no more molecular detections were performed. Hence, clone-related lesions could not be excluded [30].

These case reports highlight the rarity of these lymphomas as well as the limitation of diagnosing SLs at different anatomical sites, as usually these are classified as higher-stage lymphomas with high disease burden, and multiple sites are not biopsied unless to prove a transformation or progression in a treatment-refractory or relapse case. 

Due to the sporadic nature of this combination, there are no guidelines or consensus related to the choice of the regime. Most of the previously reported cases have received rituximab, cyclophosphamide, doxorubicin, vincristine, and prednisone (R-CHOP)-based or CHOP-based chemotherapy regimens. An R-CHOP-based regimen is most suitable, as it has a spectrum of activity against both cHL and DLBCL. It's more reasonable to target the more aggressive disease while dealing with synchronous dual primaries. While selecting the regimen, it is prudent to select a regime that is also active against lesser aggressive diseases so that good disease control can be achieved. R-CHOP is the standard of care for DLBCL, and CHOP has good activity against cHL as well. Therefore, we would have offered six doses of R-CHOP to the patient. There is no evidence of autologous or allogeneic stem cell transplant against DLBCL and CHL in the first-line treatment. So, we would have reserved autologous stem cell transplant as consolidation after salvage therapy for relapse or residual disease after primary treatment. The role of radiation is mostly limited to bulky disease in DLBCL and cHL [31].

## Conclusions

It is incredibly uncommon for Hodgkin lymphoma and B-cell lymphomas to develop simultaneously as discordant synchronous lymphomas. In light of the possibility that the incidence of discordant lymphoma may be higher than previously thought, both clinicians and pathologists need to be aware of this condition to prevent making inaccurate or overlooked diagnoses. Discordant lymphomas typically have a poorer prognosis than single lymphoma cases; therefore, the most effective therapeutic approach to treat multiple lymphomas at once is challenging, and the approach is usually based on the type of lymphoma that is more malignant. Hence, a diagnosis of discordant lymphoma, which is currently under-reported with worse patient outcomes, warrants more research to accurately determine the incidence, and its pathogenesis, and develop treatment protocols. A multidisciplinary approach with extensive workup, including immunohistochemistry, cytogenetics, and molecular studies, is necessary to reach this rare diagnosis. It is important to work up and report such cases as either individual reports, case series, or multicenter studies with larger cohorts describing the clinical, morphological, immunohistochemical, and molecular characteristics in detail, as the findings of individual cases cannot be generalized to the larger population. This would add to the existing literature and help develop diagnostic and treatment protocols as well as assess the prognosis when faced with a challenging scenario.
